# Larval Adaptation to Salinity Shock in Cane Toads ( *Rhinella marina* ) From Coastal French Guiana

**DOI:** 10.1002/ece3.72244

**Published:** 2025-10-06

**Authors:** Simon Ducatez, Rick Shine, Jayna L. DeVore

**Affiliations:** ^1^ UMR SECOPOL (IRD, IFREMER, ILM, UPF) Tahiti French Polynesia; ^2^ School of Natural Sciences, Macquarie University Sydney New South Wales Australia

**Keywords:** *Bufo marinus*, osmoregulation, phenotypic plasticity, physiological tolerance, salt, tadpole

## Abstract

Many habitats not only differ in mean conditions but also in the degree to which these conditions fluctuate through time. Therefore, local adaptation to both mean abiotic conditions and habitat variability can enhance organismal viability. Within their native range in French Guiana, cane toads ( *Rhinella marina* ) inhabit both coastal beach and inland rainforest habitats. We surveyed breeding pools to quantify abiotic conditions. Breeding pools in coastal habitats exhibited high salinity levels and intermittent seawater inflow with rapid increases in salinity. Breeding pools in inland habitats had low, stable salinity levels. Salinity shock only caused mortality within coastal environments. To determine whether larvae were locally adapted to salinity conditions, we raised the offspring of toads collected from 14 inland and coastal sites under a range of salinity levels. We also mimicked seawater inflow by exposing larvae from 15 clutches to sudden increases in salinity to measure their tolerance to salinity shock. Although coastal and inland tadpoles performed similarly under constant salinity, tadpoles from coastal habitats survived a rapid increase in salinity better than did conspecifics from inland habitats. The capacity to tolerate variable environments and adapt to local conditions may have contributed to the cane toad's translocation success.

## Graphical abstract

Many habitats not only differ in mean conditions but also in the degree to which these conditions fluctuate through time. Here, we show that native range cane toad tadpoles are adapted to local fluctuations in salinity, rather than to average salinity. Although coastal and inland populations performed similarly under constant salinity, tadpoles from coastal populations survived a rapid increase in salinity better than did conspecifics from inland habitats.

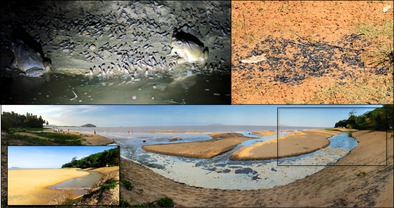

## 1 Introduction

Local adaptation can play an important role in allowing populations of a species to persist across a diversity of habitat types. Although many biotic and abiotic factors can drive local adaptation, aquatic organisms may be especially sensitive to the osmotic challenges imposed by variation in salinity (Gunter 1956; Whitehead et al. 2011) and salinity may therefore drive adaptation (Gomez‐Mestre and Tejedo 2004; Rogell et al. 2009; DeFaveri and Merilä 2014; Albecker and McCoy 2017). Organisms that can adapt to local salinity conditions may persist in times of environmental change (Albecker and McCoy 2017; Relyea et al. 2024) and colonise novel environments (e.g., during invasion). Amphibians are especially vulnerable to salinity because of their permeable skin and eggs and poor osmoregulatory abilities, with osmotic challenges causing increases in mortality, developmental deformities, and physiological stress, and altering development and growth (Shoemaker and Nagy 1977; Balinsky 1981; Katz 1989). Despite this sensitivity, more than 140 amphibian species have been recorded in saline habitats (Hopkins and Brodie 2015). Mechanisms of response to osmotic stress include tadpoles manipulating sodium and chloride concentrations within their bodies (Alvarado and Dietz 1970; Gomez‐Mestre et al. 2004) and adult amphibians increasing their osmolarity by hypersynthesizing and retaining urea (Shoemaker and Nagy 1977; Balinsky 1981; Katz 1989).

Interest in the effects of salinity on freshwater organisms has increased as human activities such as agriculture, de‐icing salt application, and resource extraction have increased wetland salinisation (Williams 2001, 2002; Cañedo‐Argüelles et al. 2013; Herbert et al. 2015; Hintz et al. 2022). Climate change and associated increases in sea level, interacting with other anthropogenic alterations, are expected to further increase the incidence and severity of saltwater intrusion and salinisation (Neubauer and Craft 2009), raising major concerns for fragile freshwater ecosystems (Cañedo‐Argüelles et al. 2013; Herbert et al. 2015; Kefford et al. 2016; Hintz and Relyea 2019). The ability of amphibians to adaptively respond to habitat salinisation remains unclear, although some species (including wood frogs, green tree frogs and natterjack toads) have evolved to tolerate high salinity levels in both coastal and roadside environments (Gomez‐Mestre and Tejedo 2003, 2004; Rogell et al. 2009; Albecker and McCoy 2017).

In contrast to tolerance to elevated salinity, amphibian tolerance to rapid changes in salinity has attracted less attention (Whitehead et al. 2011; Hopkins and Brodie 2015). As a broad generalisation, organisms often can adjust to a gradual shift in the level of an environmental factor (such as salinity, temperature or pH) via physiological acclimation. However, an abrupt transition between the same two levels can be fatal (Wu et al. 2014; Lai et al. 2019). We might therefore expect natural selection to work not only on the match between average environmental conditions and organismal tolerances but also on the ability of animals in highly variable environments to withstand sudden changes in abiotic conditions. Because dramatic variation in salinity levels is common in many osmotically challenging environments (e.g., because of tidal fluctuations, storm events or the application of road salt), an ability to withstand abrupt salinity changes may also enhance fitness. However, reactions to osmotic stress may depend on when the stressor occurs and whether exposure is transient or chronic (Alexander et al. 2012; Kearney et al. 2014). Understanding the frequency, predictability, and nature of these selection events is key to understanding adaptation in these environments (Parsons 2005; Bell 2013; Hopkins and Brodie 2015).

Animals utilise several mechanisms to deal with an unpredictable change in abiotic conditions. Among the most common are behavioural responses, whereby the organism detects and avoids dangerously extreme conditions. For example, microhabitat selection can buffer animals against extreme conditions (Scheffers et al. 2014). Those tactics are effective because of small‐scale spatial heterogeneity in levels of the threatening factor, creating “islands” of safe conditions even if average conditions have shifted into a potentially lethal range. Such heterogeneity tends to be lower in aquatic systems because the buffering effect of microhabitat selection is limited by spatial homogeneity in temperature, salinity and pH within a small waterbody. Under such conditions, the only solution is a physiological ability to deal with a sudden shift in conditions.

Isolated waterbodies provide ideal model systems with which to explore this phenomenon, especially if they differ in their exposure to abrupt changes in abiotic conditions. Some of the best examples involve waterbodies close to the ocean, where unpredictable combinations of tides, storm surge, and winds can cause periodic seawater influxes into breeding pools, whereas inland pools remain unaffected (McLean et al. 2001; Hobohm et al. 2021; Little et al. 2022). Waterbodies in French Guiana, in tropical South America, exemplify this situation. Cane toads ( *Rhinella marina* ) are widely distributed along coastal and adjacent forested regions, and spawn in small puddles, pools and ponds that range from freshwater to saline. Importantly, some of those breeding sites exhibit stable salinities, whereas others (closer to the ocean) are subject to intermittent influxes of seawater (see below). We characterised abiotic conditions and larval survival rates within a range of such ponds and used common‐garden experiments to test the ability of larval cane toads (from both coastal and forested habitats) to tolerate both varying levels of salinity and rapid changes in salinity.

## 2 Methods

### 2.1 Study Species

The cane toad ( *Rhinella marina* , formerly *Bufo marinus* ) is native to South America, but has been translocated to 138 islands and countries in the hope that it would control sugar cane pests (Lever 2001; Shine 2018). Most research on this species has been conducted within Australia, where the cane toad is a high‐profile pest and has caused major ecological impacts (Shine 2010). The biology of cane toads within their native range has attracted less scientific attention (Shine 2018). Like most bufonids, cane toads lay large clutches of small eggs in lentic (stillwater) habitats; the resulting tadpoles develop rapidly (often in < 3 weeks) and metamorphose at small sizes [< 200 mg: (Crossland et al. 2011)].

The specific name of the cane toad (“marina”) refers to its supposed ability to tolerate saltwater, on the basis of the coastal sites where the type specimens were collected in Surinam in the 1700s (Turvey 2013). Although this species typically spawns in freshwater (Zug and Zug 1979; Lever 2001; Semeniuk et al. 2007; Wijethunga et al. 2015), brackish‐water ponds are also utilised (Covacevich and Archer 1975). Tadpoles of the closely related *R. horribilis* (previously categorised within “ *R. marina* ”, (Acevedo et al. 2016)) were reported in a highly saline area in Panama (27.5% salinity: (De León and Castillo 2015)); although it is unclear whether these tadpoles survived in the long term (see Discussion). As for other amphibian species (Albecker and McCoy 2017), laboratory studies suggest that salinity tolerances of eggs and larvae in Hawai'i and Puerto Rico (to 5.25 and 8 ppt, respectively: (Ely 1944; Rios‐López 2008)) are lower than those of adults (to 14 ppt, or 40% seawater; on the basis of studies in Australia: (Liggins and Grigg 1985)). Field surveys in Australia (Hagman and Shine 2006; Semeniuk et al. 2007; Wijethunga et al. 2016) have documented relatively low salinities in breeding ponds used by cane toads (< 2.7 ppt), even at coastal sites.

### 2.2 Study Area

We conducted our fieldwork in French Guiana, one of the main source populations for most invasive cane toad populations worldwide (Slade and Moritz 1998). The climate is tropical, hot and humid throughout the year (mean monthly temp > 27**°**C throughout the year), with drier and slightly warmer weather from July to November (< 150 mm rainfall/month), and a rainy season from December to June (> 330 mm/month; BBC weather, 2019). All our observations were conducted during the dry season, between August and November 2017, as we were conducting a general survey of cane toad ecology and behaviour in their native range. Field observations were conducted opportunistically as breeding ponds were encountered, but we followed precise protocols for the experimental part of this study. In their native range, cane toads occur in a diversity of habitats, from rainforest to coastal habitats and highly disturbed urban areas (Zug and Zug 1979; DeVore et al. 2020; DeVore, Shine, and Ducatez 2021), although they generally prefer to occupy patches of open habitat during nocturnal activity (DeVore, Shine, and Ducatez 2021).

### 2.3 Field Surveys

We searched areas suitable for toad habitat in coastal, forested, and urban sites and measured salinity, pH, temperature, conductivity and total dissolved solids (TDS) in every pond in which we observed cane toad eggs or tadpoles. Measurements were taken using a digital meter 1–2 m from the pond edge (EC‐PCST Testr35, EUTECH, Singapore; accuracy ±0.01 ppt). We used a hydrometer to assess salinity when it exceeded 10 ppt (Instant Ocean SeaTest, Blacksburg, VA, USA). Details on the weather conditions during the relatively dry Aug–Nov study period are available in (DeVore, Shine, and Ducatez 2021) (Figure S1). Our aims were (1) to assess the mean water conditions of breeding pools in different habitat types, (2) to determine whether within‐pond temporal variation in water conditions differed between habitat types, and (3) to determine whether the success of tadpole clutches within breeding ponds was affected by these breeding pool parameters (i.e., salinity, habitat and size). Where possible, we repeatedly measured the same ponds to assess the stability of these conditions across time, sampling the same pond up to 10 times. Out of the 18 breeding ponds that we identified, seven were sampled twice or more (Table 1). Because urbanisation is known to alter the salinity of freshwater streams and water bodies (Kaushal et al. 2018; Estévez et al. 2019), we considered urban habitats as a separate category. As a result, our habitat variable included three categories: urban, non‐urban coastal (< 7 km from the coast; hereafter, “coastal”) and non‐urban inland (> 25 km from the coast; hereafter “rainforest”).

**TABLE 1:** Salinity within cane toad breeding ponds and associated cane toad clutch outcome in French Guiana. Salinity means ± SE; range in brackets. Although salinity measurements within coastal breeding pools occasionally reached 35 ppt following tidal inundation, live tadpoles were only observed at < 6 ppt (measurements below were therefore also taken at times when tadpoles were absent). Cane toad tadpoles were periodically present in all monitored ponds; 24 of these clutches were also monitored in order to determine the proportion of naturally deposited clutches that successfully produced metamorphs, as well as identify the causes of clutch collapse. Five additional coastal rock pools that contained cane toad tadpoles (e.g., Figure 1b) are excluded, as they dried before salinity measurements were taken.

Pond	# samples	Salinity (ppt)	# monitored clutches	# successful clutches	Causes of failure	Latitude	Longitude
Coastal							
Beach/rock outcrop	3 ponds 17 measurements	7.03 ± 4.06 [0.11; 35]	8	5	2 tidal influx 1 drying		
Gosselin pond	10	10.96 ± 4.49 [0.11; 35]	4	3	1 tidal (35 ppt)	4.8907	−52.2529
Montjoly rock pool 1	5	8.25 ± 6.7 [0.52; 35]	4	2	1 tidal (35 ppt) 1 drying	4.9133	−52.2599
Montjoly rock pool 2	2	1.89 ± 0.23 [1.66; 2.12]				4.9124	−52.2584
Anthropogenic	2 ponds 2 measurements	0.19 ± 0.07 [0.13; 0.26]					
Kourou marina pool	1	0.13				5.1519	−52.6758
Cayenne drainage pond	1	0.26				4.8948	−52.3300
Inland							
Rainforest	13 ponds 18 measurements	0.03 ± 0.01 [0.02; 0.07]	16	8	7 drying 1 overcrowding		
Bélizon wetland	1	0.02				4.3396	−52.5142
East road pool 1	1	0.02				4.4909	−52.3466
East road pool 2	2	0.03 ± 0.01 [0.02; 0.04]	2	2		4.4909	−52.3466
East road pool 3	2	0.07 ± 0.01 [0.06; 0.07]	1	1		4.4909	−52.3466
East road pool 4	1	0.02				4.4909	−52.3466
East road pool 5	1	0.03	1	0	1 drying	4.4909	−52.3466
East road pool 6	2	0.02 ± 0.001 [0.02; 0.022]	1	0	1 drying	4.4909	−52.3466
East road pool 7	2	0.02 ± 0.01 [0.02; 0.035]	3	2	1 drying	4.4909	−52.3466
Kaw lumberyard pool	1	0.03	1	0	1 drying	4.5490	−52.1719
Past Regina wetland	1	0.02				4.2027	−52.1258
Regina wash pool	1	0.02				4.3634	−52.2799
Regina wash left pool	1	0.02	2	0	1 drying, 1 overcrowding	4.3640	−52.2788
Steep bank marsh	2	0.02 ± 0 [0.02; 0.02]	2	2		3.9568	−51.8592
Kaw pond			1	1		4.6437	−52.2991
Kaw puddle			1	0	1 drying	4.6437	−52.2991
St Georges pool			1	0	1 drying	3.9457	−51.8504

To determine whether breeding pool parameters affected clutch success, we tracked a total of 24 clutches of naturally deposited eggs across a range of habitat types. Each egg mass or tadpole school observed in an independent waterbody was considered as one clutch. In some waterbodies, several clutches were observed across time (e.g., if eggs were later observed in the same pond where tadpoles were present earlier, they were considered as a new clutch). We considered a clutch as successful if any individuals within the clutch reached metamorphosis, and as a failure if the entire clutch died prior to that time (mortality was evident if all tadpoles were observed to be dead or if no tadpoles or metamorphs were present < 4 days after observing early‐stage tadpoles). Therefore, only clutches whose fate could be reliably assessed were included, as we did not conduct a systematic and regular monitoring of all observed clutches. We identified three causes of these mortality events: pond drying, seawater influx and extremely high tadpole densities that apparently led to clutch collapse. Flooding due to high rainfall also swept some additional monitored clutches out of their breeding pools into nearby stream systems, but we excluded these clutches from consideration as we could not determine whether the tadpoles had subsequently survived.

### 2.4 Laboratory Experiments

In order to produce egg clutches, we collected adult cane toads from 14 sites ranging from 0–51 km from the coast. The toads were kept in perforated 100 * 40 * 15 cm plastic containers with wood‐chip bedding. Each container hosted four to eight toads, depending on their size (keeping males and females separate), as well as a water container and two black plastic boxes open on one side to serve as shelters. The containers were cleaned every 3 days, and the toads were fed with cockroaches on a daily basis. Temperature was maintained at 26°C, and we used the natural day‐night light cycle.

Within 1 week of capture, we randomly paired adult toads by site, and added each pair to an inclined 100*40*15 cm perforated plastic container partly filled with freshwater (at 0.034 ppt). We injected the two toads with leuprorelin acetate to stimulate oviposition between 1700 h and 2000 h (see ref (Hayes et al. 2009) for details) and collected the eggs the next morning. The eggs were kept in containers of the same size as the breeding ones, filled with ~10 liters of freshwater (0.034 ppt), in the same temperature and light cycle conditions as the adults, until the larvae reached stage 25. We added air pumps to the egg containers to keep the water oxygenated, and changed half of the water on a daily basis. Upon reaching stage 25 (i.e., the free‐swimming, feeding tadpole stage, reached after about 5 days), tadpoles from nine clutches (three from inland habitats (≥ 25 km from the coast), six from coastal habitats [< 7 km from the coast]) were haphazardly selected for inclusion in Experiment 1. We also raised additional tadpoles from 15 clutches (10 from inland habitats, 5 from coastal habitats) for use in Experiment 2. All tadpoles were housed at a salinity of 0.034 ppt prior to being used in the experiments.

### 2.5 Experiment 1: Survival, Growth and Development at Different Salinity Levels

When the eggs had developed into free‐swimming tadpoles [Gosner stage 25; the stage at which feeding begins (Gosner 1960)], tadpoles from nine separate clutches were transferred to 1‐L containers filled with 500 mL of water (0.034 ppt). Five tadpoles were added to each container, and we used three replicates per treatment per clutch, except for one clutch where only two replicates were included (because of the low number of tadpoles available from that clutch). Salinity levels were then adjusted gradually to 0.034, 1, 4 or 8 ppt within each container, beginning with 25% mixtures of water of the final salinity concentration (with 75% 0.034 ppt water) for the first 8 h, followed by 50% mixtures for the following 16 h. This 24 h acclimation period was included to reduce salinity shock, as 24 h of acclimation to intermediate salinity has been found to enhance salt tolerance in other anurans (Lai et al. 2019). After 24 h, the water was changed to reach the target salinity concentration. In the first two clutches, no tadpoles survived at 8 ppt after 12 h, whereas all tadpoles had survived at salinity levels of 1 and 4 ppt. We therefore modified our protocol to expose the subsequent seven clutches to additional intermediate salinity treatments: 5, 6, and 7 ppt. Thus, two clutches were exposed to salinities of 0.034, 1, 4 and 8 ppt, and seven clutches were exposed to salinities of 0.034, 1, 4, 5 (1 clutch), 6, 7 and 8 ppt. Tadpoles were fed ad libitum daily with ground algae pellets (Hikari Algae Wafers, Kyorin Co. Ltd., Himeji, Japan) at which time survival was assessed (with the first check occurring 12 h after the target salinity was reached). Instant Ocean Sea Salt (Blacksburg, VA, USA) was used to adjust water salinity. Tadpoles were held at 26°C, and complete water changes were performed every 3 days. Ten days after the experiment commenced, tadpoles were euthanized (with MS222: Argent Chemicals, Redmond, WA, USA) and measured (mass after blotting dry and Gosner stage). For one coastal clutch, we only obtained survival data because of logistical constraints. All available data were used in all analyses.

### 2.6 Experiment 2: Effect of Salinity Shock on Short‐Term Survival

We exposed tadpoles from 10 clutches produced by parents from inland habitats and five from coastal areas to a salinity shock to measure their short‐term survival after a rapid increase in salinity. This experiment was conducted simultaneously for all 15 clutches (5 to 20 days post laying). This treatment aimed to mimic an abrupt change in salinity similar to those we had documented when a spring tide causes seawater to flow into coastal ponds. For each clutch, we used 15 tadpoles previously raised at a salinity of 0.034 ppt, weighed them to assess mean tadpole mass, and transferred five individuals directly into 500 mL of water at 0.034 ppt, 1 ppt, or 8 ppt. We then counted the number of surviving tadpoles in each treatment after 12 h.

### 2.7 Analyses

#### 2.7.1 Field Surveys

To assess whether mean salinity differed between habitat types, we compared the salinity measurements of each breeding pool. We excluded time periods where no eggs or tadpoles were present. Where repeated measures were taken over multiple days, we averaged these measurements to calculate one mean value per waterbody. Salinity could also vary considerably within different areas of a waterbody (e.g., between 2.51 and 8.47 ppt). Where multiple measures were taken on the same day, all measurements were averaged to obtain a single value for that day. We then assessed whether mean salinity varied as a function of habitat (or distance from the coast) using separate linear models to compare the mean parameters of these waterbodies.

To determine whether clutch success was significantly affected by waterbody parameters, we tested possible predictors of whole‐clutch survival for the 24 naturally laid clutches within 13 monitored waterbodies, including distance from the coast (km), salinity (ppt) and waterbody size (m^2^). The success of each clutch within the waterbody was considered as a binomial outcome and modelled using logistic regression with a quasibinomial error to correct for data overdispersion (dispersion parameter = 1.3).

#### 2.7.2 Experiment 1

To assess the effect of prolonged exposure to different salinity levels on tadpole survival, we built a generalised linear mixed model with survival (proportion of surviving tadpoles per container following 10 days of exposure) as the response variable and habitat, salinity and the interaction between habitat and salinity as explanatory variables. Clutch identity was included as a random factor. As survival was expressed as a proportion, we used a logit link function specifying a quasibinomial error to correct for data underdispersion (deviance/residual df = 0.46). Using the mean value per container to avoid pseudoreplication, the effects of salinity manipulation on rates of growth (mass, mg) and development (Gosner stage) were analysed using linear mixed models (LMM). Either body mass or developmental stage after 10 days of treatment was used as the response variable, and we included the effects of salinity, habitat and their interaction as fixed explanatory variables. Clutch identity was included as a random factor. We then used stepwise selection to eliminate non‐significant variables (*p* < 0.05). Statistics are provided for each variable, considering the last model before they were discarded for non‐significant effects, and the final model for significant effects. Our data on body mass and developmental stage were log‐transformed to meet assumptions of homoscedasticity. In all cases, results were consistent whether parental habitat was considered as a category (i.e., coastal vs. rainforest) or a continuous predictor (km from the coast), so here we present the results of the categorical predictor for simplicity.

#### 2.7.3 Experiment 2

To assess the effect of an abrupt increase in salinity on tadpole survival, we built a generalised linear mixed model with survival (proportion of live tadpoles per container at 8 ppt) as the response variable and habitat, body mass and the interaction between habitat and body mass as explanatory variables. Clutch identity was included as a random factor. As survival was expressed as a proportion, we specified a quasibinomial error to correct for overdispersion (dispersion parameter = 2.06). All tadpoles survived at 0.034 ppt and 1 ppt, so these two levels were not included in the analysis.

## 3 Results

**FIGURE 1:**
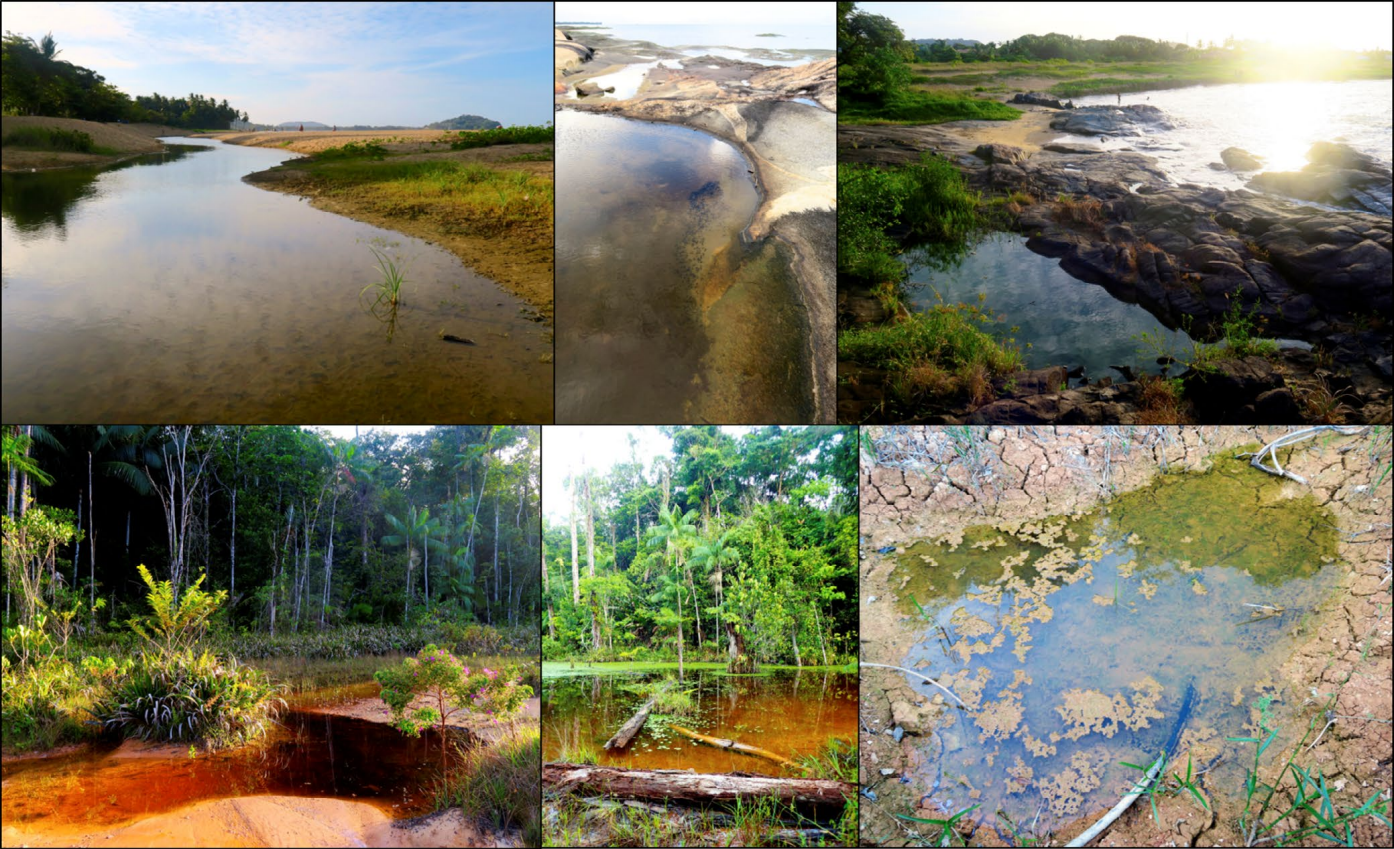
Breeding sites of cane toads ( *Rhinella marina* ) in French Guiana, in coastal (upper panels) and inland rainforest (lower panels) habitats.

**FIGURE 2:**
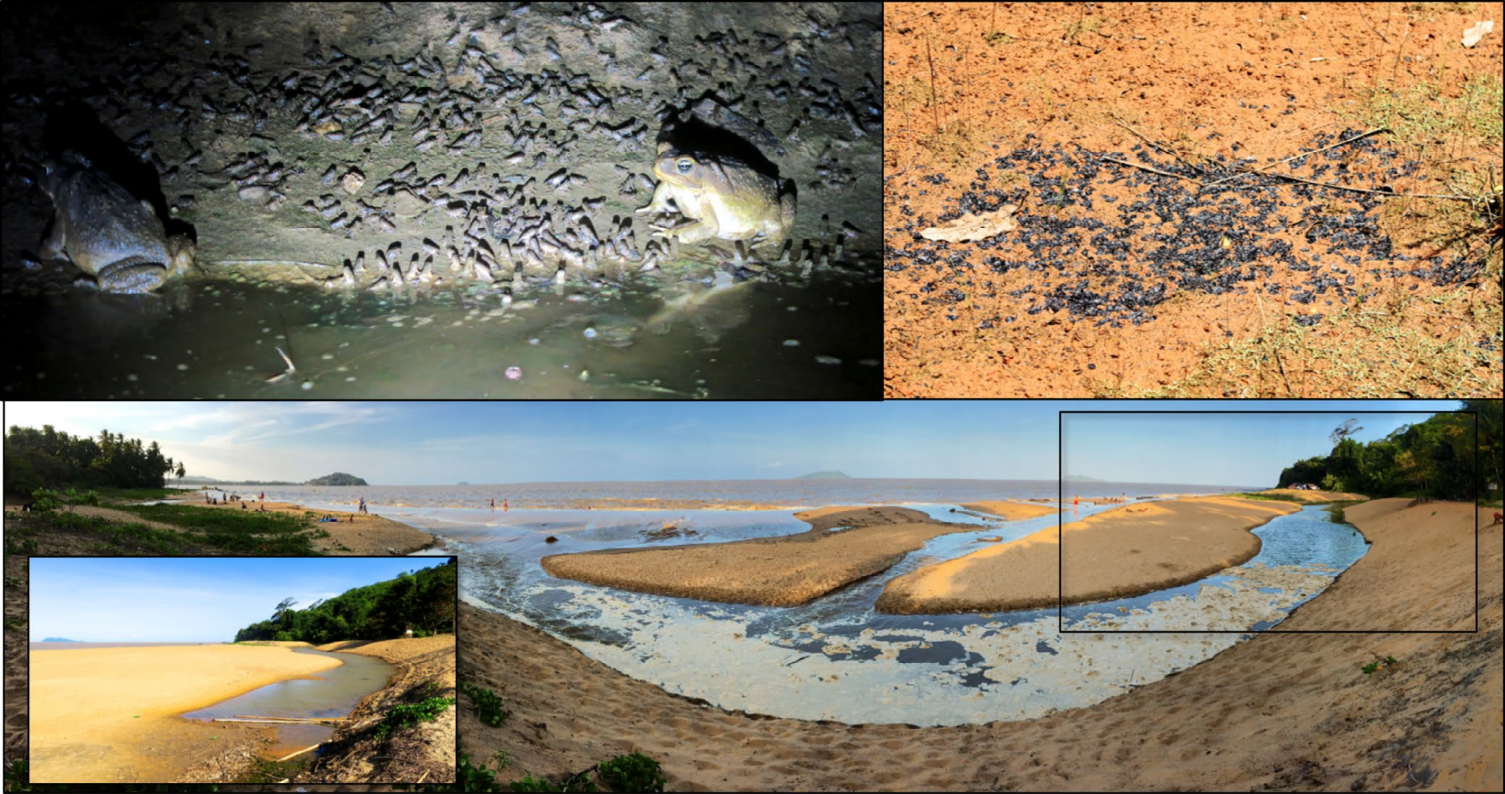
Illustration of cane toad clutch outcome and causes of failure in French Guiana. Clutches were considered successful if metamorphs were produced (upper left, salinity: 0.218 ppt). Causes of whole‐clutch failure included pond drying (upper right) and seawater influx (lower panel: The photo inset shows the waterbody, containing thousands of tadpoles, prior to tidal inundation [1.089 ppt]. After inundation, salinity exceeded 30 ppt and no tadpoles survived.) Cane toads preferentially breed in temporary waterbodies, and metamorphosis can be rapid; 27 days prior to the metamorphosis documented in the upper left, this waterbody was too saline for egg or tadpole development (22 ppt).

Along the coast, adult cane toads breed [and rehydrate; (DeVore, Shine, and Ducatez 2021)] in pools separated from the sea by sand banks, as well as in rock pools that form on outcrops of the Guianan shield (Figure 1). These ponds are regularly impacted by both rainfall (that can rapidly decrease breeding pool salinity) and tidal fluctuations or waves that periodically bring varying quantities of seawater (Figure 2). Inland, cane toads bred in temporary rain puddles formed after heavy rain events, open marshes, and pools formed by river drying (Figure 1). In urban areas, human‐made ponds and ditches were also utilized by breeding toads.

**FIGURE 3:**
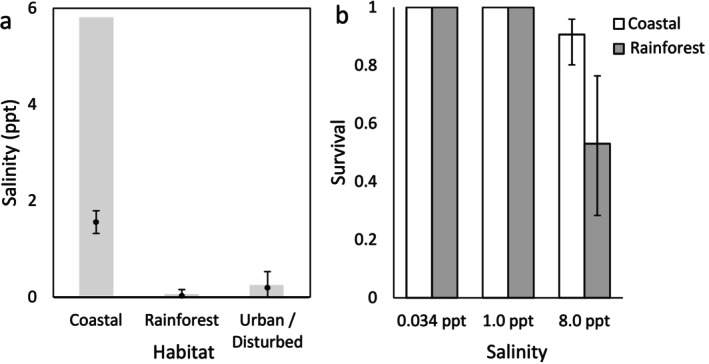
Variation in salinity across breeding ponds and differences in tadpole survival in response to a sudden salinity increase in cane toads from different habitats in French Guiana. Along the coast, tadpoles were not only found in higher mean salinity conditions than in rainforest or urban habitats (panel a; points show means ± SE) but were also found within a greater range of salinity conditions (grey bars). When exposed to a sudden change in salinity in the lab (0.034 to 8 ppt), tadpoles produced by parents from coastal habitats (< 7 km from coast, 5 clutches) were more likely to survive than were those from inland rainforest habitats (≥ 25 km from coast, 10 clutches, *p* = 0.0499, panel b, means ± SE). The probability of survival also increased with tadpole mass (*p* = 0.0478). All tadpoles survived transfers to water with the same (0.034 ppt) or similar (1 ppt) salinities.

At times when tadpoles were present, mean salinity differed between pools from different habitat types (chisq = 32.626, df = 2, *p* = 8.23E‐8); the salinity of coastal breeding pools was higher than in rainforest (Tukey post hoc: 1.533 ± 0.27 (SE), df = 16, *t* = 5.686, *p* = 0.0001) or urban (1.364 ± 0.408 (SE), df = 16, *t* = 3.341, *p* = 0.0109) breeding pools (Figure 3a). Mean salinity did not differ significantly between rainforest and urban habitats (*t* = −0.471, *p* = 0.886). In addition, salinity was relatively stable both across time and space in the four rainforest ponds (with means ranging between 0.02 and 0.07 ppt; Table 1) and two anthropogenic ponds (0.13 and 0.26 ppt). Salinity could also vary substantially both over time and between coastal ponds (e.g., between 0.11 and 35 ppt or 0.52 and 35 ppt, Table 1). However, high salinity levels were lethal, with salinity levels > 30 ppt associated with periodic whole‐clutch mortality events, and the maximum salinity level at times when live tadpoles were observed to persist for > 12 h was 5.82 ppt. In two cases, we observed the moment at which seawater influxes rapidly raised the salinity levels to > 30 ppt; although tadpoles survived short‐term in these conditions (live tadpoles were still observed when revisited 2–3 h later, with most clustering in shallow water along the pond edges), all tadpoles were dead when the waterbodies were revisited the following day.

Pond conductivity and total dissolved solids followed the same pattern as salinity, whereas mean pH and temperature of breeding pools did not differ significantly between habitat types (range: 5.65–7.76, 24°C–36.6°C; Table 1).

Causes of whole‐clutch mortality in 24 naturally‐laid clutches included pond drying (8 clutches) and tidal influx (2 clutches). In addition, the tadpoles of one clutch were found floating dead in a small and shallow (< 3 cm deep) water puddle, covering the entirety of the puddle surface. Overcrowding may have caused the whole clutch to collapse (potentially via starvation followed by anoxia). Overall, whole‐clutch mortality during the study period was 46%, but the probability of clutch failure did not differ significantly between habitat types (chisq = 0.2685, df = 1, *p* = 0.6043) nor, alternatively, with distance from the coast (chisq = 0.0909, df = 1, *p* = 0.7630). Neither mean salinity (chisq = 0.0566, df = 1, *p* = 0.8120) nor waterbody area (chisq = 0.07725, df = 1, *p* = 0.7811) significantly predicted the probability of clutch failure.

### 3.1 Experiment 1: Survival, Growth, and Development at Different Salinity Levels

**FIGURE 4:**
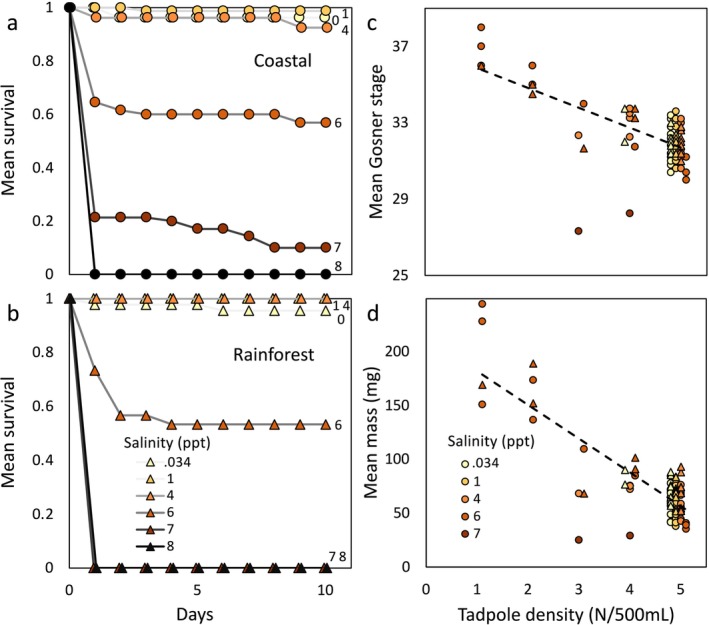
Effects of salinity treatment on cane toad tadpole survival, mass and development. The rates of development and survival of tadpoles were affected by salinity treatment during the 10‐day rearing period but were not significantly influenced by parental origin. Panels a and b depict mean daily tadpole survival across clutches produced by six coastal (a) and three inland (b) pairs during a 10‐day rearing period at each salinity level (143 tanks containing 5 tadpoles each). Panels c and d depict the significant effects of final tadpole density on rates of tadpole development (c) and tadpole growth (d). Here, each point represents the mean performance of all individuals within a tank at a given salinity level, where tadpole density is the number of individuals remaining within the tank. Tadpoles that survived within the 6 ppt treatment benefited from reduced tadpole densities within this treatment, ultimately developing and growing more quickly than in high‐density, low‐salinity conditions. Note that only 7 sibling tadpoles from 2 tanks survived in the 7 ppt treatment, and the development and growth of these individuals were lower than that within any other treatment. Round symbols are used to designate clutches produced by parents collected in coastal environments (< 7 km from the coastline), whereas triangles represent clutches produced by inland parents (> 25 km from the coastline).

Tadpoles were less likely to survive at higher salinity compared to lower salinity (estimate = −1.263 ± 0.423; DF = 124; *t* = −2.982; *p* = 0.003): no tadpole survived at 8 ppt, only 8% (i.e., 7 individuals from one clutch) survived at 7 ppt, 56% survived at 6 ppt, and 95 to 99% at 4, 1 and 0.034 ppt. In contrast, the interaction between salinity and habitat (estimate = −0.272 ± 1.081; *t* = −0.251; DF = 123; *p* = 0.802) and the main effect of habitat (forest vs. beach; estimate = −1.115 ± 1.915; DF = 7; *t* = −0.582; *p* = 0.579) did not significantly affect larval survival. Most (90%) tadpole mortality occurred in the first 12 h after tadpoles were exposed to their target salinity levels (Figure 4). The number of surviving tadpoles in the tank affected the mean mass (mg) and developmental stage of tadpoles after 10 days. In replicates with fewer surviving tadpoles (i.e., lower densities), survivors had a higher mean mass and more advanced developmental stage (mass: estimate = −0.233 ± 0.020; t = −11.618; DF = 81; *p* < 0.0001; Gosner stage: estimate = −0.0262 ± 0.0033; t = −7.951; DF = 81; *p* < 0.0001). Neither salinity (mass *p* = 0.254; Gosner stage: *p* = 0.106), habitat (mass *p* = 0.338; Gosner stage *p* = 0.799), nor their interaction (mass *p* = 0.978; Gosner stage *p* = 0.570) affected mass or developmental stage; these factors were removed from the final model. These effects on survival, growth and development remained consistent if distance from the coast, rather than habitat type, was considered as a predictor. If salinity was considered as a categorical rather than a continuous predictor, the effect of density remained, but salinity also significantly affected both development and growth; both were significantly reduced in the 7 ppt treatment relative to other salinity levels (Tukey posthoc; *p* < 0.0001 in all cases). All other posthoc comparisons were not significant.

### 3.2 Experiment 2: Effect of Salinity Shock on Short‐Term Survival

All tadpoles exposed to a salinity of 1 ppt survived, as did the control tadpoles that were maintained at 0.034 ppt. We therefore focused our analyses on survival at 8 ppt. The interaction between habitat and body mass did not significantly affect post‐exposure tadpole survival (estimate = −0.0009 ± 0.0078; *z* = −0.112; *p* = 0.9130) and was excluded from the model. However, both body mass (estimate = 0.004 ± 0.002; *z* = 2.204; *p* = 0.0478) and habitat (estimate = −1.309 ± 0.601; *z* = −2.180; *p* = 0.0499) significantly affected larval survival. Coastal tadpoles were more likely to survive a sudden increase in salinity from 0 to 8 ppt (Figure 3), and survival probability increased with body mass.

## 4 Discussion

In the field, cane toads within the native range were found breeding over a wide span of salinities. These salinities varied between habitats, such that breeding pools in coastal habitats had both higher mean salinity levels and greater variation in salinity across time. However, despite higher mean salinities in coastal breeding habitats, low‐salinity conditions were also encountered in all habitat types. Ultimately, it was the ability to cope with variation rather than mean differences that apparently differed between coastal and inland tadpoles, with tadpoles produced by parents from coastal habitats showing a greater tolerance to salinity shock than did those from inland habitats.

Although cane toad tadpoles can tolerate a wide range of salinity conditions, this tolerance is not exceptional among anurans (Hopkins and Brodie 2015). Here we found that, despite some early mortality, tadpoles from every clutch tested could develop normally at (or below) 6 ppt, but consistently either died or suffered from severe retardation of growth and development at 7 and 8 ppt. Although tadpole survival was lower at 6 ppt than at lower salinities, early mortality in this treatment, followed by high survival, growth and development rates, implies a failure to acclimate to changing salinity conditions (Wu et al. 2014) or a greater salinity sensitivity in early development (see (Alexander et al. 2012)) was responsible for this effect. Individuals that survived for the first 2 days at 6 ppt grew and developed as well as in lower salinity treatments; in fact, individuals raised at 6 ppt actually grew and developed more quickly overall, because of the early reduction in density that apparently favoured rapid growth (if the effect of density is removed, tadpoles developed and grew significantly faster in 6 ppt water than at 0.034, 1, 4 or 7 ppt; Tukey post hoc *p* < 0.0005 in all cases after removing the number of surviving tadpoles from the model). Density was therefore a more important predictor of tadpole growth and development than was salinity. Conspecific density is well known as an important driver of tadpole performance, and its effects can overwhelm those of abiotic variation or local adaptation (Albecker et al. 2020). Ultimately, we found no evidence of local adaptation in salinity tolerances in coastal vs. inland habitats; cane toad tadpoles are apparently tolerant of relatively high salinities even in environments 50 km from the coast where breeding pool salinities were consistently low, and tadpoles from coastal habitats performed as well as those from inland habitats in low‐salinity conditions.

This salinity tolerance within the native range is similar to that experimentally documented in invasive populations. For example, for cane toad egg clutches in Hawaii, metamorphosis occurred in salinities of 3.5 and 5.25 ppt (10, 15% seawater); mortality 3 days post‐hatch occurred in 7 ppt (20% seawater), and complete mortality was documented at or before hatching in 8.75 and 17.5 ppt [25, 50% seawater; (Ely 1944)]. However, cane toad tadpoles from invasive inland populations in Puerto Rico exhibited a higher salinity tolerance than was documented here, successfully reaching metamorphosis at 0, 1, 2, 4 and 8 ppt but dying at 12 ppt (Rios‐López 2008). In invasive Australian populations, tolerance to high salinity conditions has not been tested, but tadpole survival was negatively affected by low salinity, with lower survival in 0.040 than at 0.13, 0.44, 0.84 or 1.23 ppt water (Wijethunga et al. 2015). This negative effect of very low salinity is consistent with our findings in the native range, as survival was lower in the 0.034 ppt treatment than at 1 ppt (chisq = 20.27, df = 1, *p* < 0.0001; although high in both cases: 96 vs. 99%; a similar pattern has also been detected in other species, calling for more work on the underlying mechanisms (Chinathamby et al. 2006; Kearney et al. 2014)). Although survey work in the invasive range has documented a narrower range of salinity within breeding pools than we documented here (0–2.7 ppt vs. 0.0171–5.82) (Hagman and Shine 2006; Semeniuk et al. 2007; Wijethunga et al. 2016), invasive cane toads have also been anecdotally reported to breed in tidal flats and brackish water in Australia and New Guinea (Covacevich and Archer 1975). Similarly, previous surveys of native‐range breeding pools have only documented toads breeding at a low mean salinity [0.135 ppt ± 0.087 SE; Venezuela (Evans et al. 1996)], but anecdotal reports of toads inhabiting exposed mudflats (which led to the original naming of this species as *Rhinella marina* [“marine toad”] by Linnaeus) imply that this species has long been thought capable of surviving saline conditions (Turvey 2013). Previous native‐range surveys that excluded coastline environments may have underestimated the full range of salinity conditions experienced by cane toad tadpoles.

One outlier to these previous observations is a report of late‐stage tadpoles and metamorphs at 27.5 ppt in the closely related species *R. horribilis* within its native range on Coiba Island, Panama (De León and Castillo 2015). Although this observation implies that these tadpoles can develop in much higher salinities than have been otherwise documented, we note that this observation occurred during a spring tide on a day when tide levels were higher than they had been for 27 days (20 Feb 2015; Balboa station, Panama). This timing raises the possibility of a similar monthly pattern of tidal inundation of breeding pools in Panama as in French Guiana, and that salinity levels may have been lower before this observation was made. As this was a single observation, it is also unclear whether these tadpoles survived long‐term. Although we also documented live tadpoles in > 30 ppt water in the hours following seawater influxes, none of these tadpoles survived until the following day. Because much of our understanding of salinity tolerances is based on single observations of live animals in the field (Hopkins and Brodie 2015), the fact that salinity levels can vary considerably over short periods is an important consideration when calculating the conditions suitable for tadpole development.

Our finding that tolerance to salinity shock increases with tadpole mass implies that tadpoles may better withstand salinity increases late in development, extending the period for which coastal waterbodies can support tadpole development. Here, no early‐stage tadpoles (stage 25) survived at 8 ppt, despite a 24 h acclimation period, but 70% of older tadpoles survived a rapid transfer to 8 ppt, and larger tadpoles were more likely to survive this transfer. Adult amphibians commonly tolerate higher salinities than do tadpoles and eggs (Albecker and McCoy 2017), but the potential for ontogenetic shifts in tolerance within a life stage has attracted less attention (Alexander et al. 2012; Kearney et al. 2014). Frequent salinity fluctuations within coastal habitats may determine how long these waterbodies remain suitable for tadpole development, imposing strong selection for the ability to tolerate periodic saltwater incursion. More generally, an ability to survive temporary or long‐term osmotic stress is likely to be favoured in these unstable environments (Kearney et al. 2014).

Although we found no evidence of local variation in tolerance of mean salinity levels across habitats, we did find that coastal tadpoles were more tolerant than inland tadpoles to a rapid increase in salinity. As our field data demonstrate that salinity fluctuations can be both rapid and fatal in coastal breeding pools, the ability to acclimate quickly to altered conditions is likely adaptive in these habitats. Note that a rapid decrease in salinity may also be ecologically relevant (e.g., following rain events), but we did not test the potential effects of rapid salinity decreases. The possibility that high salinity levels could persist only briefly means that even short‐term tolerance to high salinity could be adaptive, even if such events cause carry‐over effects with sub‐lethal effects on future performance (Kearney et al. 2014). Our data on whole‐clutch failure in the field also highlight the disadvantages of breeding in temporary waterbodies; whether because of drying or salinity shock, nearly half of the naturally deposited clutches that we monitored failed to produce any metamorphs. Although temporary waterbodies may provide a refuge from competition and predation, these high clutch failure rates highlight the critical importance of rapid development. Notably, our study species resembles many other Bufonid anurans in its relatively brief larval period. This difference between habitats in tolerance to a salinity shock calls for future investigations of the underlying mechanism (e.g., genetic, epigenetic and parental effects).

Extensive research on cane toads within their invaded range in Australia has documented rapid evolutionary change in a wide range of phenotypic traits, driven by a combination of mechanisms including adaptive evolution, phenotypic plasticity and spatial sorting (Shine et al. 2011; Stuart et al. 2019; DeVore, Crossland, et al. 2021). Some of the traits that have shifted involve physiological mechanisms that adapt local populations to specific abiotic challenges, such as heightened risk of desiccation (Kosmala et al. 2020) or low ambient temperatures during activity periods (McCann et al. 2017). Habitat‐specific tolerance of salinity shock, as demonstrated in the current study, adds an example of a different trait—and importantly, adds an example from the native range of the cane toad. These data demonstrate that cane toads already exhibited an ability for adaptation to site‐specific abiotic challenges before they were translocated across the world. Such a capacity has been expressed along multiple dimensions during the toads' invasion of Australia, plausibly enhancing the species' success in colonising environments that lie well outside the “climate envelope” within the native range (Tingley et al. 2014). The roots of the cane toad's conquest of Australia, thus, may include evolutionary pressures for the ability to make rapid adaptive adjustments to deal with unpredictable abiotic challenges within the species' native range.

## Author Contributions

**Simon Ducatez:** conceptualization (equal), data curation (equal), formal analysis (equal), investigation (equal), methodology (equal), project administration (equal), software (equal), validation (equal), visualization (equal), writing – original draft (equal), writing – review and editing (equal). **Rick Shine:** conceptualization (equal), funding acquisition (equal), supervision (equal), writing – review and editing (equal). **Jayna L. DeVore:** conceptualization (equal), data curation (equal), formal analysis (equal), investigation (equal), methodology (equal), project administration (equal), software (equal), validation (equal), visualization (equal), writing – original draft (equal), writing – review and editing (equal).

## Conflicts of Interest

The authors declare no conflicts of interest.

## Supporting information


**Figure S1:** Map of French Guiana showing the position of the breeding ponds monitored during this study (white arrows) together with the sites where adults used in the experimental approach were collected (white circle with central black dot).

**Table S1:** ece372244‐sup‐0001‐TableS1.xlsx.

**Table S2:** ece372244‐sup‐0002‐TableS2.xlsx.

## Data Availability

All data used in this manuscript are available as Supporting Information or in Table 1.
