# Supplementary figures and images for: Larval Adaptation to Salinity Shock in Cane Toads ( Rhinella marina ) From Coastal French Guiana
Source: Ecol Evol. 2025 Oct 6;15(10):e72244. doi: 10.1002/ece3.72244 (PMC12498011; doi:10.1002/ece3.72244)

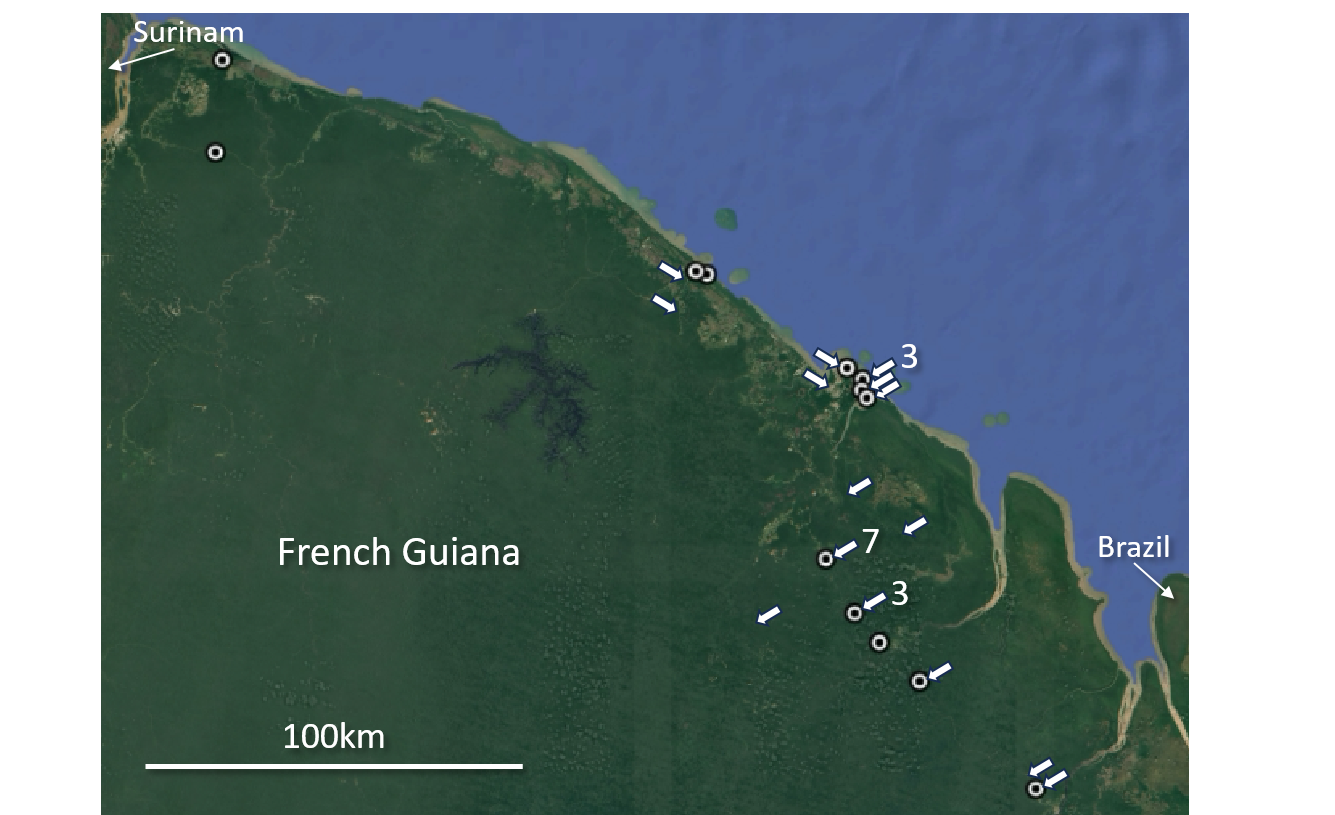

Supplement: Supplementary file 1 — Figure S1: Map of French Guiana showing the position of the breeding ponds monitored during this study (white arrows) together with the sites where adults used in the experimental approach were collected (white circle with central black dot). [file ECE3-15-e72244-s003.tif]
